# EMR1/ADGRE1 Expression in Cancer Cells Upregulated by Tumor-Associated Macrophages Is Related to Poor Prognosis in Colorectal Cancer

**DOI:** 10.3390/biomedicines10123121

**Published:** 2022-12-02

**Authors:** Rokeya Akter, Kwangmin Kim, Hye Youn Kwon, Youngwan Kim, Young Woo Eom, Hye-mi Cho, Mee-Yon Cho

**Affiliations:** 1 Department of Pathology, Wonju College of Medicine, Yonsei University, Wonju 26426, Republic of Korea; 2 Department of Surgery, Wonju College of Medicine, Yonsei University, Wonju 26426, Republic of Korea; 3 Department of Colorectal Surgery, Wonju College of Medicine, Yonsei University, Wonju 26426, Republic of Korea; 4 Regeneration Medicine Research Center, Wonju College of Medicine, Yonsei University, Wonju 26426, Republic of Korea

**Keywords:** colorectal cancer, adhesion G protein-coupled receptor EMR1/ADGRE1, tumor-associated macrophages, macrophage activation, lymph node metastasis, recurrence-free survival

## Abstract

EMR1, a member of the adhesion G protein-coupled receptor family (ADGRE1), is a macrophage marker that is abnormally expressed in cancer cells. However, its clinical significance in colorectal cancer (CRC) is not well-known. In this investigation, EMR1 expression in tumor cells (EMR1-TC) was found in 91 (22.8%) of the 399 CRC samples tested by immunohistochemical staining and showed a significant relationship with lymph node metastasis. Furthermore, EMR1-TC was significantly associated with CD68^+^ CD163^+^ tumor-associated macrophages (TAMs), and CRC with a high combined EMR1-TC^+^CD68^+^CD163^+^ score showed worse recurrence-free survival prognosis. In an in vitro co-culture assay of colon cancer cells with myeloid cells, we found that EMR1 expression significantly upregulated in cancer cells was induced by macrophages. In addition, there was increased expression of M2 markers (CD163 and interleukin-6 & 10) in myeloid portion, while that of M1 markers (CD86 and iNOS) remained unchanged. Accordingly, upon treatment with M2 macrophage polarization inhibitors (O-ATP, trametinib, bardoxolone methyl), EMR1 expression reduced significantly, along with M2 markers (CD163 and interleukin-6 & 10). In conclusion, EMR1-TC was a high-risk factor for lymph node metastasis and correlated with poor recurrence free survival, particularly in patients with TAM-rich CRC. Furthermore, EMR1 expression in colon cancer cells may be related to M2 macrophage polarization and vice versa.

## 1. Introduction

Colorectal cancer (CRC) is the most prevalent cancer and the third leading cause of cancer-related mortality worldwide [1,2]. Patients with progressive CRC have poor clinical outcomes, despite application of modern surgical methods, adjuvant/neoadjuvant chemotherapy, and molecular target treatments [3]. Therefore, to improve patient outcomes, it is important to identify new innovative treatment targets for CRC.

The tumor microenvironment (TME) is a special biological environment formed by a heterogeneous group of cells, including leukocytes, cells of the myeloid lineage, fibroblasts, endothelial cells, and their secreted components [4]. This complex microenvironment can support tumor growth and progression by protecting the tumor from host immune reactions, promoting therapeutic resistance, and providing niches for metastasis [5]. Macrophages are one of the most prevalent types of immune cells in the TME [6]. They have two distinct functional subtypes: M1, which inhibits tumor growth by producing pro-inflammatory cytokines [7,8], and M2, which promotes tumor growth by producing anti-inflammatory cytokines [9,10]. The majority of tumor-associated macrophages (TAMs) have characteristics of M2-polarized cells [11,12]. In particular, microsatellite unstable (MSI-H) CRC has more immune cell infiltration, including tumor-infiltrating lymphocytes and macrophages, than microsatellite stable (MSS) CRC; these tumor-infiltrating immune cells play a vital role in the prognosis of MSI-H CRC [13,14,15]. Several studies have shown that macrophage-rich CRC is associated with high lymph node metastasis (LNM) and is indicative of poor survival [16,17]. Contradictory information has also been reported; that is, a high number of infiltrating macrophages correlates with a better prognosis for patients with CRC [18,19]. Therefore, the clinical significance of macrophage infiltration in CRC remains elusive.

Epidermal growth factor (EGF)-like module-containing mucin-like hormone receptor-like 1 (EMR1/ADGRE1) is an orphan receptor that belongs to the epidermal growth factor-seven-transmembrane subfamily of group-II adhesion G protein-coupled receptor (aGPCRs) [20]. EMR1, also known as F4/80, is a well-established pan-macrophage marker in mice, and has recently been used as a marker for myeloid cells in humans, such as eosinophils and macrophages [21,22]. It is inducible during monocyte–macrophage differentiation from progenitors in the bone marrow and can act as a modulator of immune cell function [21,22,23]. Several studies have used EMR1 as a TAM marker [24,25,26], while others described it as a prognostic biomarker in various cancers [16,17,18,19]. Soncin et al. reported that intra-tumoral EMR1-positive macrophages support colon cancer progression [27]. Furthermore, EMR1 expression in tumor cells was also reported in several carcinomas, including CRC, and has a strong correlation with immune cell infiltration in TME [20,28,29,30]. Lei et al. demonstrated that EMR1/ADGRE1 expression in cancer exhibited a positive correlation with immune cell infiltration in uterine corpus endometrial carcinoma [31]. Ali et al. also suggested that the abnormally expressed EMR1 in CRC manipulates the immune system [32]. However, as of yet, the possibility of EMR1 as a prognostic factor and its clinicopathological correlation with CRC remains unclear.

Therefore, in this study, we aimed to determine the clinical significance of EMR1 expression in CRC in association with TAM. In addition, we investigated the co-effect of EMR1 expression in colon cancer cells (CCs) and macrophage polarization in vitro.

## 2. Materials and Methods

### 2.1. Patients and Tissue Samples

In this study, we used formalin-fixed and paraffin-embedded tissues from 399 surgically resected CRC samples (71 MSI-H and 328 MSS) registered at the Wonju Severance Christian Hospital BioBank, Wonju, Republic of Korea. Patients who had received neoadjuvant chemo and/or radiotherapy were excluded from the study. Furthermore, MSI-L CRC cases were also excluded because the case numbers were not sufficient for statistical analysis. Moreover, some excluded cases consisted of those with unknown MSI status.

### 2.2. Ethics Approval

The Institutional Ethics Committee of Yonsei University, Wonju College of Medicine, granted the approval for this study (approval no.: CR-321336), which was carried out in accordance with the Declaration of Helsinki.

### 2.3. Immunohistochemical (IHC) Staining

IHC staining of the paraffin-embedded tissue sections was performed as previously described [33]. After examining the hematoxylin and eosin-stained slides of each case, we selected a region that was 3 mm in size from the center of the tumor. We then obtained the tissue core from the paraffin block to produce a tissue microarray block. A 4-μm tissue section of the tissue microarray paraffin block was attached to a coated slide, following which immunostaining was performed using an automatic staining machine (BenchMark XT, Ventana Medical Systems, Tucson, AZ, USA). The slides were incubated for 2 h with primary antibodies against pan-macrophage marker EMR1 (Cell Signaling Technology, Danvers, MA, USA), TAM marker CD68 (Abcam, Cambridge, UK) and M2 macrophage marker CD163 (Abcam, Cambridge, UK) at 37 °C, in an autostainer using an UltraView Universal DAB Detection Kit (BenchMark XT, Tucson, AZ, USA). The slides were then examined under an Olympus BX51 microscope (Olympus, Tokyo, Japan). On evaluation of the dyeing results, CD163 and EMR1, which were expressed in both cancer cells (TC) and inflammatory cells (SC), were graded according to the percentage of positive cells in each as follows: 1 (0 or <5%), 2 (6–25%), 3 (26–50%), 4 (51–75%), and 5 (>75%). The final immunoreactive score was classified as high (≥3 = 2) or low (≤3 = 1). CD68 was only expressed in inflammatory cells, and the positivity rate was analyzed using an Image analyzer (GenASIs HiPath, Applied Spectral Imaging, Tel Aviv, Israel)). The combined expression of EMR1 in tumor cells (EMR1-TC), CD68, and CD163 was scored as high (8) and low (1, 2, 4) according to the results of multiplying each score.

### 2.4. Cell Culture

#### 2.4.1. Cell Culture and Reagents

The human monocyte cell line, THP-1, and CRC cell lines (HCT15 and Caco2) were obtained from the Korean Cell Line Bank. The cells were grown in Roswell Park Memorial Institute (RPMI)-1640 medium (HyClone, Logan, UT, USA) containing 10% fetal bovine serum (FBS), 1% penicillin (100 U/mL) and streptomycin (100 mg/mL; Gibco, Grand Island, NY, USA), at 37 °C, in a humid environment with 5% CO_2_.

#### 2.4.2. Co-Culture Procedures

Cell culture inserts (0.4-m pores; Corning, NY, USA) were used for the co-culture system. We adopted the macrophage polarization protocol from our previous study [34]. First, THP-1 monocytes (1 × 10^6^ cells/mL) were seeded into the top chamber of a Transwell^®^ plate. The THP-1 cells were then exposed to 200 nM phorbol-12-myristate-13-acetate for 48 h to induce differentiation and generate M0 macrophages. Further, the cells were cultivated for 24 h, after washing twice with RPMI medium to eliminate the phorbol-12-myristate-13-acetate interference. The culture supernatant collected thereafter was labeled M0 macrophage-conditioned medium (M0-CM). To polarize M1 or M2 macrophages, THP-1 cells were treated with phorbol-12-myristate-13-acetate for 48 h, following which M1-polarizing reagents (100 ng/mL lipopolysaccharide + 20 ng/mL interferon; R&D Systems, Minneapolis, MN, USA) or M2-polarizing reagents (20 ng/mL IL-4 + 20 ng/mL IL-13; R&D Systems, Minneapolis, MN, USA) were added to the cells. These were then incubated at 37 °C, for 48 h. The culture supernatant collected was labeled M1 macrophage-CM (M1-CM) or M2 macrophage-CM (M2-CM).

To facilitate cell adhesion, HCT15 (1 × 10^4^ cells/mL) and Caco2 (1 × 10^6^ cells/mL) cells were seeded in the lower chamber and incubated for 24 h. THP-1 and THP-1-derived macrophages (M0, M1, and M2) were then immediately positioned on top of a six-well plate containing HCT15 and Caco2 cells, and the resulting co-culture system was used to cultivate the cells for 48 h (except HCT15-M1-coculture, which followed 72 h incubation) in RPMI-1640 medium. CRC-THP1 cells were co-cultured with or without M2 macrophage inhibitors (62.5 μM O-ATP, Sigma Aldrich, St. Louis, MO, USA, 2.5 μM Trametinib; Selleckchem, Houston, TX, USA) and M1/M2 macrophage inhibitor (312.5 nM bardoxolone methyl, Selleckchem, Houston, TX, USA) for 48 h. After incubation, both the upper and lower chamber cells were lysed for mRNA and protein analysis. As controls, HCT15 and Caco2 cells, as well as THP-1 and THP-1-derived macrophages (M0, M1, M2), were cultured for 48 h in a six-well plate containing RPMI-1640 medium.

#### 2.4.3. Flow Cytometry Analysis

Trypsinized cells were centrifuged and resuspended in FBS Stain Buffer (BD Pharmingen™, San Diego, CA, USA). Phycoerythrin (PE)-conjugated IgG1 k (isotype control) and EMR1-PE monoclonal antibody (Santa Cruz Biotechnology, Santa Cruz, CA, USA) were added to each group and incubated in the dark for 60 min at 4 °C. After washing, the cells were resuspended in FBS Stain Buffer for analysis with a BD FACS Aria III (BD Bioscience, San Jose, CA, USA).

#### 2.4.4. RNA Extraction and cDNA Synthesis

Following the manufacturer’s instructions, total RNA was extracted from the co-culture system using TRIzol^®^ Reagent (Invitrogen, Waltham, MA, USA) and the isopropanol–chloroform technique. To make cDNA, 1 μg of total RNA was reverse-transcribed using the QuantiTect^®^ Reverse Transcription Kit (Qiagen, Hilden, Germany) with gDNA wipeout buffer (7×), RT primer mix, and 1 μL of Quantiscript Reverse Transcriptase (200 units), in a final volume of 20 μL, for 15 min at 42 °C, followed by a 3 min denaturation at 95 °C.

#### 2.4.5. Real-Time Quantitative Reverse Transcription Polymerase Chain Reaction (qRT-PCR) mRNA Assay

qRT-PCR was carried out in 384-well PCR plates with a final volume of 10 μL, using Fast SYBR^®^ Green Master Mix (Applied Biosystems, Waltham, CA, USA), cDNA template, and RT forward and reverse primers. Triplicate reactions were set up for each primer/cDNA pair. Glyceraldehyde 3-phosphate dehydrogenase (GAPDH) served as the internal control. Real-time PCR reactions were initiated in a QuantStudio™ 6 Flex System (Thermo Fisher Scientific, Waltham, MA, USA) by heating to 50 °C for 2 min and then to 95 °C for 10 min, followed by 40 cycles of 95 °C (15 s) and 60 °C (60 s). Relative quantification of gene expression was performed using the threshold cycle (C_t_) method.

#### 2.4.6. Western Blot

The proteins were separately prepared from the cells. The cells were lysed in a sodium dodecyl sulfate–polyacrylamide gel electrophoresis (SDS–PAGE) sample buffer (62.5 mM Tris-HCl (pH 6.8), 1% SDS, 10% glycerol, and 5% β-mercaptoethanol). The proteins were boiled for 5 min, subjected to SDS–PAGE, and transferred to an Immobilon polyvinylidene difluoride membrane (Millipore, Burlington, MA, USA). The membrane was blocked with 5% skim milk in Tris-HCl buffered saline containing 0.1% Tween 20 and then incubated with primary antibodies against EMR1 (F4/80) and anti-β-actin (1:1000, Santa Cruz Biotechnology, Santa Cruz, CA, USA), followed by peroxidase-conjugated secondary antibodies (1:2000, Santa Cruz Biotechnology, Santa Cruz, CA, USA). The membrane was then treated with EZ-Western Lumi Pico (DOGEN, Seoul, Korea) and visualized using the ChemiDoc XRS+ system (Bio-Rad, Hercules, CA, USA).

#### 2.4.7. Statistical Analysis

All statistical analyses were performed using SPSS (version 25.0; IBM, New York, NY, USA) and Prism (version 8.0, GraphPad Software, La Jolla, CA, USA). Chi-square test or Fisher’s exact test was used to compare categorical data provided as frequencies and percentages. Spearman’s correlation analysis and Pearson’s chi-square test were used to examine the relationships between all variables. A forward step-wise selection of variables was used to perform a multivariable logistic regression analysis. The results of one- and two-way analyses of variance group test were used to assess the in vitro co-culture variables, which were represented as the mean with standard deviation. The Kaplan–Meier technique was utilized for survival analysis and generation of survival curves, while the log-rank test was performed to quantify the differences between patient subgroups. Univariate and multivariate Cox regression analyses were used to identify independent prognostic variables. In all cases, statistical significance was set at *p* < 0.05.

## 3. Results

### 3.1. Association of EMR1-TC with Clinicopathological Variables in Patients with MSI-H and MSS CRC

A total of 399 patients with CRC who underwent colorectal surgery with curative intent were enrolled in this study, among which 219 patients had received 5-Fluoruracil-based adjuvant chemotherapy after surgery. Follow-up duration was a minimum of 0.3 months and a maximum of 178.5 months (median 42.1 months). These included 71 cases of MSI-H and 328 of MSS CRC. Of these, 368 patients (92.2%) were more than 50 years in age and 31 patients (7.8%) were less than or equal to 50 years in age; 155 (38.8%) were female and 244 (61.2%) were male; 227 (56.9%) had lymph node non-metastasis (LN^−^) and 172 (43.1%) had lymph node metastasis (LN^+^). MSI-H cancer was located more frequently in the right- than left-sided colon (13.8% vs. 4.0%), while MSS was more common in the left- than right-sided colon (52.6% vs. 29.6%).

**Figure 1:**
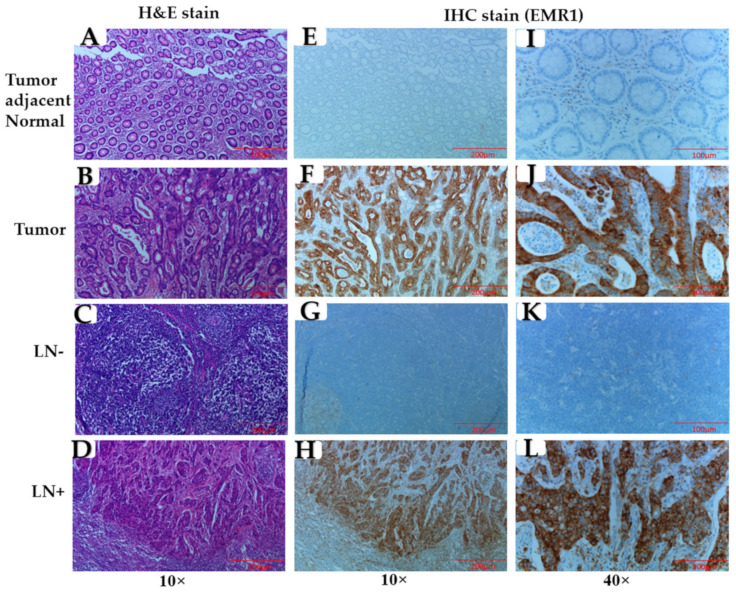
EMR1 expression in primary tumor and lymph node (LN) of colorectal cancer. (**A–D**) Colon tissues stained with hematoxylin and eosin (H&E); and (**E**–**L**) EMR1 immunohistochemistry (IHC) staining. (**A**,**E**,**I**) tumor adjacent normal area; (**B**,**F**,**J**) tumor area; (**C**,**G**,**K**) non-metastasis lymph node, LN^−^; and (**D**,**H**,**L**) metastasis lymph node, LN^+^. Total number of samples, *n* = 399. Total number of samples used for representative images, *n* = 3. Original magnification, 10× and 40×.

Upon analyzing the clinicopathological significance of EMR1-TC in MSI-H and MSS CRC, EMR1-TC pattern was concordant in primary tumors and matched positive lymph nodes, as compared to tumor-adjacent normal colon and LN^−^ tissues (representative images in Figure 1A–L). The relationships between EMR1-TC and clinical variables are summarized in Table 1. Of the 399 patients with CRC, EMR1-TC was almost 2-fold higher in MSI-H than in MSS CRC (38.0% vs. 19.9%, *p* < 0.003). EMR1-TC was significantly correlated with tumor stage (T_stage, *p* = 0.044), LNM (*p* = 0.001), and lymphatic invasion (*p* = 0.030) in MSI-H CRC, while MSS CRC showed significant correlation only in LNM (*p* = 0.037) among all clinicopathological parameters. However, upon considering total CRC (MSI-H + MSS), EMR1-TC showed a significant relationship with tumor location (*p* = 0.003), T_stage (*p* = 0.039), and LNM (*p* = 0.003). Pearson’s chi-square and Spearman’s rho correlation (bivariate) analyses revealed that EMR1-TC was positively correlated with LNM in CRC (Table 1 and Table S1). Upon carrying out multivariate logistic regression analysis, we found that LNM occurrence with high EMR1-TC expression was 10.18-fold higher in MSI-H [hazard ratio = 10.18, 95% confidence interval (C.I.) = 2.344–44.244, *p* < 0.002] and 1.98-fold higher in MSS CRC [hazard ratio = 1.98, 95% confidence interval (C.I.) = 1.11–3.54, *p* < 0.021] than with low expression (Table 2).

### 3.2. Correlation between EMR1-TC and CD68^+^/CD163^+^ TAMs in MSI-H and MSS CRC

**Figure 2:**
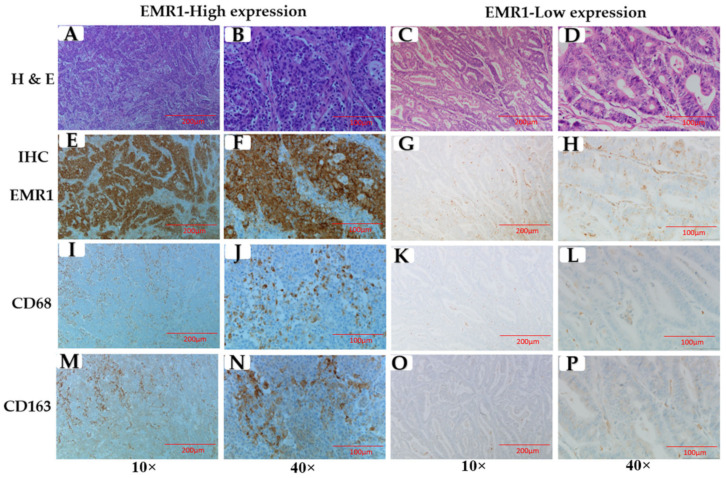
EMR1, cluster of differentiation (CD)68, and CD163 expression in primary tumor of colorectal cancer (CRC). (**A–D**) Colon cancer tissues stained with hematoxylin and eosin (H & E), (**E–H**) EMR1, (**I–L**) CD68, and (**M–P**) CD163 immunohistochemistry (IHC) staining in primary tumor of CRC. CD68^+^CD163^+^ TAMs were higher in high EMR1-TC expression area compared to low expression area (**E–P**). Total number of samples, *n* = 399. Total number of samples used for representative images, *n* = 2. Original magnification, 10× and 40×.

We observed that CD68^+^ and CD163^+^ TAM density was significantly higher in the high EMR1-TC area than in the low area (Figure 2A–P). EMR1-TC was positively correlated with EMR1 expression in stroma cells (EMR1-SC) and CD68^+^ and CD163^+^ TAMs in the MSI-H and MSS CRC groups independently, as well as in total CRC (MSI-H+MSS) cases (Table 1 and Table S1). Although individual CD68^+^ and CD163^+^ TAMs were insignificantly correlated with LNM, the combined EMR1-TC^+^CD68^+^CD163^+^ score was significantly correlated with LNM (Table 3). In the multivariate logistic regression analysis, we observed that the expression levels of CD68^+^ (7-fold) and CD163^+^ (4-fold) TAMs were significantly higher in tissues with high EMR1-TC expression than in those with low expression, especially in MSI-H CRC (Table 2). These results suggest that EMR1-TC in CRC has a strong relationship with CD68^+^CD163^+^ TAMs and plays a vital role in the LNM of CRC.

### 3.3. Survival Analysis of Combined EMR1-TC^+^CD68^+^CD163^+^ Expression in Patients with MSI-H and MSS CRC

We analyzed the prognostic significance of a combined EMR1-TC^+^CD68^+^CD163^+^ expression and clinicopathological features in patients with CRC.

**Figure 3:**
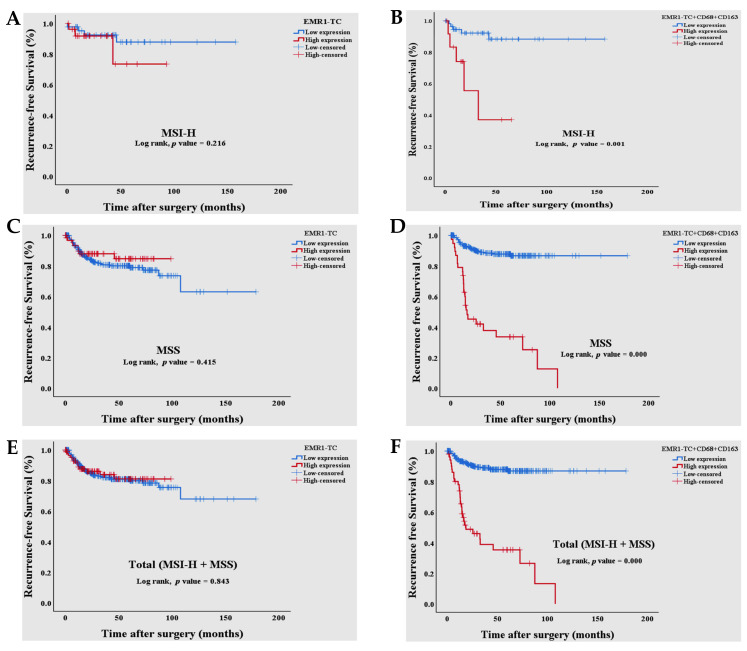
A high combined EMR1-TC^+^CD68^+^CD163^+^ expression illustrated the poor RFS in CRC. (**A**,**C**,**E**) Kaplan–Meier curves showed that there was no significant difference between high EMR1-TC expression compared to low expression; (**B**,**D**,**F**) However, high combined EMR1-TC^+^CD68^+^CD163^+^ expression was significantly associated with worse RFS in MSI-H and MSS CRC compared to low combined expression. The *p*-value was obtained using the log-rank test of the differences. Statistical significance was set at *p* < 0.05. Abbreviations: CD, cluster of differentiation; CRC, colorectal cancer; EMR1-TC, EMR1 expression in tumor cells; MSI-H, microsatellite unstable; MSS, microsatellite stable; RFS, recurrence-free survival.

Univariate and multivariate Cox regression as well as Kaplan–Meier survival analysis showed that the high combined EMR1-TC^+^CD68^+^CD163^+^ expression was significantly associated with worse recurrence-free survival (RFS) in MSI-H and MSS CRC (Table 4 and Figure 3A–F). RFS odd was 6.565-fold higher in patients with a high combined EMR1-TC^+^CD68^+^CD163^+^ expression than in patients with low expression [hazard ratio (HR) = 6.565, 95% C.I. = 3.988–10.806, *p* < 0.000]. T_stage, lymphatic invasion, and LNM were also significantly related to RFS in patients with CRC.

### 3.4. Macrophage-Induced EMR1 Upregulation in Colon Cancer Cells (CCs) In Vitro

**Figure 4:**
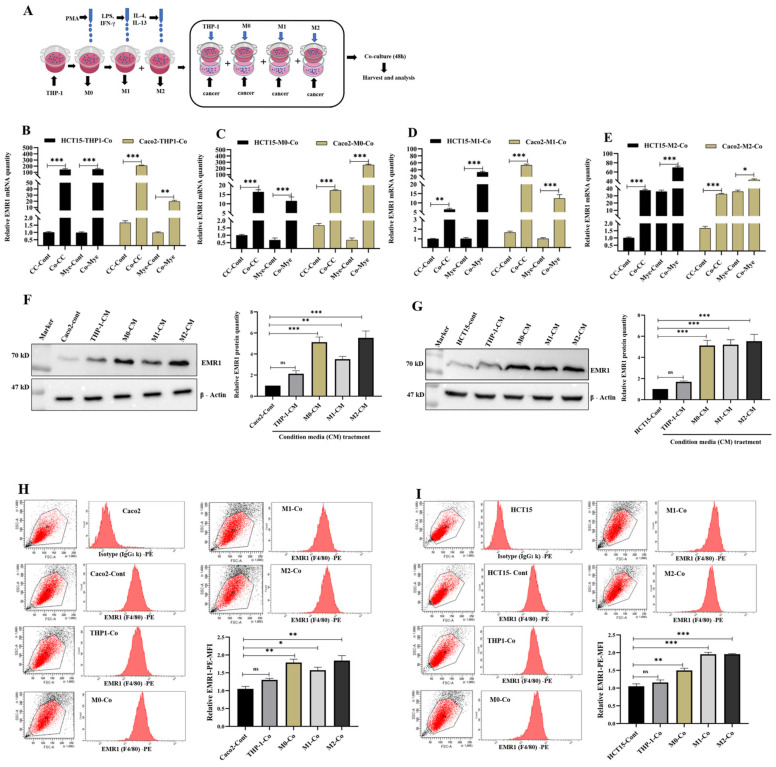
High EMR1 expression in colon cancer cells was induced by macrophages. (**A**) Schema for representing the experimental procedures. (**B**–**E**) Relative EMR1 mRNA level was detected in both colon cancer (HCT15, Caco2) and myeloid (THP1-monocyte, M0, M1, M2 macrophages) cells co-cultured for 48 h as determined by real-time quantitative reverse transcription polymerase chain reaction. Results were normalized by GAPDH. (**F**,**G**) Relative EMR1 protein expression was detected in both HCT15 and Caco2 cancer cells after 48 h of CM treatment produced from myeloid cells (THP-1monocyte, M0, M1, M2 macrophages), as determined by western blot analysis. (**H**,**I**) Flow cytometry analysis also showed increased EMR1 expression in colon cancer cells (Caco2, HCT15) after being co-cultured with myeloid (THP-1, M0, M1, M2 macrophage) cells for 48 h. Error bars denote SD. *Statistically significant at * *p* < 0.033; ** *p* < 0.002; *** *p* < 0.001, in comparison with the control group through multiple Bonferroni one- and two-way ANOVA group test. Abbreviations: CC-cont, colon cancer cell-control; CM, conditioned media; Co, co-culture; Mye-cont, myeloid cell-control; SD, standard deviation, MFI, Median fluorescence intensity.

High EMR1 expression in CCs was induced by macrophages in an in vitro co-culture experiment. The real-time quantitative reverse transcription polymerase chain reaction (qRT-PCR) analysis was performed to analyze the EMR1 expression after co-culture of CCs and myeloid cells (THP-1 monocytes, M0, M1, and M2 macrophages) separately in a non-contact Transwell^®^ system; this system enabled the interchange of soluble substances but was impermeable to the cells themselves (Figure 4A). After 48 h of co-culture, there was a significant increase in EMR1 mRNA levels in both CCs and myeloid cells, compared to that in the control (Figure 4B–E and Figure S1). Apart from the treatment with THP-1-CM, treatment with macrophage (M0, M1, and M2)-derived CM also significantly induced higher EMR1 protein expression in both HCT15 and Caco2 cells, compared to that in the control (Figure 4F,G). The flow cytometry analysis also showed that CCs with EMR1 expression increased after co-culturing with myeloid cells, compared to that in the control (Figure 4H,I). However, in the CC-THP1 co-culture group, we observed a significant increase in EMR1 mRNA levels in both the CCs and myeloid portion, compared to those in the control, indicating that after being co-cultured, THP1-monocytes are possibly differentiated into macrophages that influence EMR1 expression in CCs. These results suggest that high EMR1 expression in CCs is influenced by unknown soluble factors released from macrophages.

### 3.5. EMR1 Upregulation in Cancer Cells Correlates with M2 Macrophage Polarization

**Figure 5:**
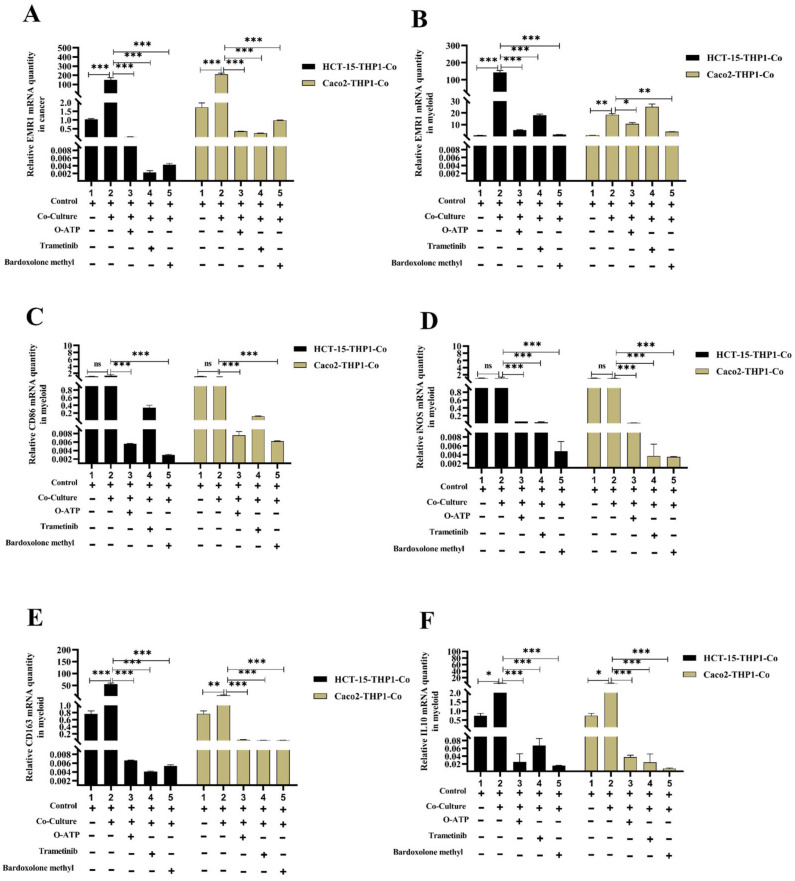

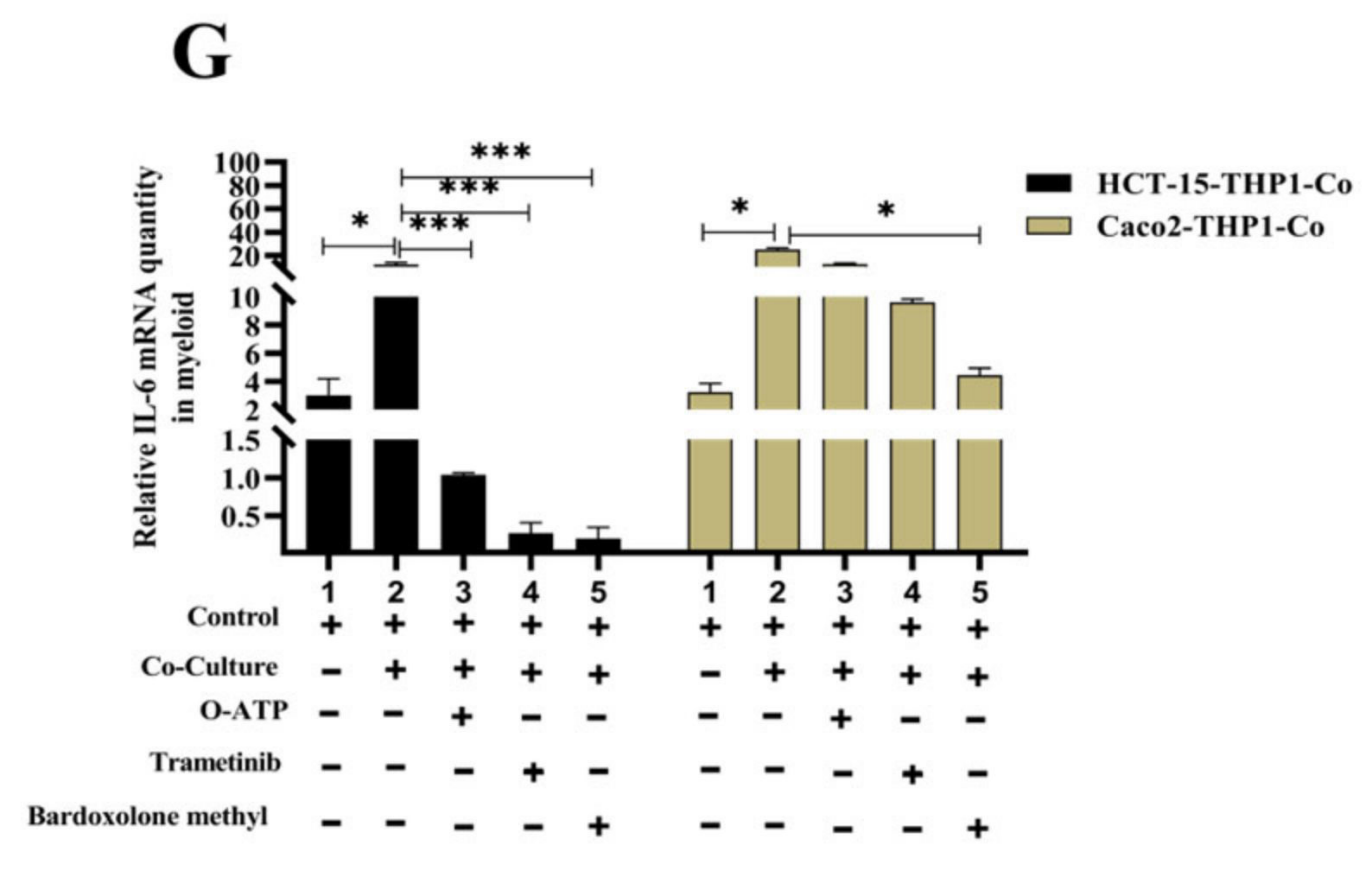
High EMR1 expression in cancer cells was correlated with M2 macrophage polarization in colon cancer. (**A**,**B**) Relative EMR1 mRNA level was detected in both CCs (HCT15, Caco2) and THP1-monocyte portion after being co-cultured with or without M2 macrophage inhibitors (O-ATP, Trametinib, and Bardoxolone methyl) for 48 h, as determined by real-time quantitative reverse transcription polymerase chain reaction; (**C**,**D**) Relative mRNA level of M1 macrophage markers (CD86 and iNOS), (**E**) M2 macrophage marker CD163, and (**F**,**G**) Cytokines interleukin (IL)-10 and IL-6 were detected in THP1-monocyte portion after being co-cultured with or without M2 macrophage inhibitors. Results were normalized by GAPDH. Error bars denote standard deviation. The results are representative of three independent experiments. * Statistically significant at * *p* < 0.033; ** *p* < 0.002; *** *p* < 0.001 in comparison with the control as well as co-cultured group by multiple Bonferroni two-way ANOVA group test.

Furthermore, we examined the effect of high EMR1 expression of CCs on macrophage polarization. In the CC-THP1 co-culture group, there was a significant increase in the expression of the M2 macrophage marker CD163, and cytokines interleukin (IL)-6 and IL-10 in the myeloid portion, along with an increase in EMR1 expression, while the expression of the M1 markers CD86 and inducible nitric oxide synthase (iNOS) remained unchanged. On the contrary, when M2 macrophage polarization inhibitors (O-ATP, trametinib, and bardoxolone methyl) were added to the co-culture system, there was a significant decrease in CD163, IL-6, and IL-10 mRNA levels in the myeloid portion, along with a decrease in EMR1 expression in both cancer and myeloid portions (Figure 5A–G). The treatment concentration of the macrophage polarization inhibitors (O-ATP, trametinib, and Bardoxolone methyl) did not affect the cell viability (Figure S2A–C). These results suggested that EMR1 expression in CCs may induce M2 macrophage polarization and vice versa in the in vitro co-culture experiments.

## 4. Discussion

First, we explained that EMR1-TC was associated with CD68^+^CD163^+^ TAMs and related to LNM and RFS in patients with CRC. We also suggested that macrophage polarization is related to EMR1 expression in CCs and vice versa.

EMR1, also known as adhesion G protein-coupled receptor ADGRE1, is a surface receptor that belongs to the epidermal growth factor-seven-transmembrane family of group-II aGPCRs, and is expressed in myeloid cells, such as monocytes, macrophages, Kupffer cells, eosinophils, and basophils [20,23]. G protein–coupled receptors (GPCRs) are the largest superfamily of membrane receptors in humans. Based on their seven transmembrane-spanning (7TM) domains, GPCRs are classified into five families: glutamate, rhodopsin, adhesion, frizzled/taste, and secretin. The aGPCRs subfamily has 33 receptors, which are divided into 8 groups. They are highly expressed in various cells and are essential for signal transduction, immune response, and cell motility, growth, and adhesion [28]. In contrast, dysregulation of several aGPCRs is associated with the development of various diseases. Given that more than 30% of all medications, including a number of anticancer drugs, have been developed to target GPCRs [35,36], it is important to investigate and comprehend the clinical significance, biological function, and molecular mechanisms of GPCRs in malignant tumors. According to several studies, solid tumors exhibit alterations in the expression of GPCR subtypes. CD97/ADGRE5 is highly elevated in breast, thyroid, stomach, pancreatic, esophageal, and colorectal cancers, and has been linked to tumor metastasis through the β-catenin, PI3K/AKT, MAPK, and Rho-GEFE signaling pathways [37,38,39,40,41,42]. GPR56/ADGRG1 is upregulated in CRC tissues and cell lines and promotes tumor growth and metastasis by inducing epithelial-to-mesenchymal transition [43]. GPR116/ADGRF5 plays a critical role in promoting breast cancer progression and metastasis through the p63RhoGEF-RhoA/Rac1 signaling pathway [44]. High ELTD1/ADGRL4 expression is linked to LNM and poor outcomes in patients with CRC [45]. However, the Cancer Genome Atlas reported EMR1 expression in CRC but did not consider it as a prognostic factor. In the current study, we reported that EMR1-TC was significantly upregulated in 22.8% of patients with CRC and was related to LNM in clinical samples. In LN^+^ cases, EMR1-TC in the primary site and LNM followed a similar expression pattern; when EMR1-TC was high in the primary tumor, it was also high in the LNM. However, because of unidentified ligands, the regulatory molecular mechanism, downstream signal transduction pathways, and genes of EMR1 in tumors that can assist LNM are still not well-known.

In the current study, we observed a correlation between EMR1-TC and CD68^+^/CD163^+^ TAMs; EMR1-TC was a high-risk factor for tumor recurrence, especially in patients with macrophage-rich CRC. Many previous studies have indicated that tumor-promoting factors affect TAM differentiation and play important roles in cancer progression by stimulating tumor growth, invasion, metastasis, and immune suppression [46]. Some studies found that CD163^+^ or CD68^+^ macrophages were associated with LNM and poorer overall survival (OS) and RFS [16,17,47,48]. Likewise, several studies showed that a high number of infiltrating macrophages correlated with lower tumor grade, less lymph node metastasis, and a better prognosis for patients with CRC [49,50,51,52]. However, the relationship between EMR1-TC and CD68^+^/CD163^+^ TAMs has not been investigated before. It is assumed that once EMR1 engages with its ligand, it participates in intracellular signaling events that lead to cytokine production and further facilitate TAM polarization and differentiation in CRC progression. To the best of our knowledge, this is the first study to reveal that EMR1-TC is correlated with CD68^+^/CD163^+^ TAM expression and serves as a high-risk factor for tumor recurrence in macrophage-rich CRC.

In our study, we enrolled 71 MSI-H and 328 MSS CRC cases and excluded MSI-L CRC cases because the numbers were insufficient for statistical analysis. MSI-H CRC has more immune cell infiltration, including tumor-infiltrating lymphocytes, than MSS CRC, and those tumor-infiltrating immune cells play a vital role in the prognosis of MSI-H CRC [13,14,15]. Tumor-infiltrating macrophages are also higher in MSI-H than in MSS CRC [53,54]. Lin et al. reported that EMR1 is required for the induction of efferent CD8^+^ regulatory T lymphocytes [55], but the relationship between EMR1-TC and CD68^+^/CD163^+^ TAMs has not been revealed before. We found that EMR1-TC was more correlated with CD68^+^/CD163^+^ TAMs than with CD3^+^/CD8^+^ tumor-infiltrating lymphocytes, and this relationship was more significant in the tumor center than in the tumor-invasive area (data not shown). Our univariate and bivariate analyses revealed that EMR1-TC was significantly correlated with CD68^+^CD163^+^ TAMs and LNM in the MSI-H and MSS CRC groups independently (Table 1 and Table S1). Moreover, in the multivariate logistic regression analysis, we observed that EMR1-TC was significantly correlated with CD68^+^CD163^+^ TAMs rather than MSI status (data not shown).

Recent meta-analyses and research studies reported a favorable prognosis with higher immune infiltration at the invasive front of tumors [56,57] in contrast to the non-invasive front [58,59]. In our study, we selected the tumor center area for evaluation because the TAMs in the invasive front showed substantial bias for the examiner in the preliminary experiment (data not shown). The univariate and multivariate analyses revealed that individual expression of EMR1-TC, CD68, and CD163 in tumor centers could not notably predict RFS or OS (Table 4 and Table S2), but combined EMR1-TC^+^CD68^+^CD163^+^ expression in the tumor center area significantly correlated with worse RFS in CRC. Although EMR1 can be an unfavorable prognostic biomarker in macrophage-rich CRC, more studies would be needed to identify the unknown ligand and signaling pathway of EMR1, which may provide further insights into how EMR1 is activated and related to LNM and RFS.

In the co-culture experiment portion of this study, we found that EMR1 expression significantly increased in all CCs, and M1 and M2 macrophages. Interestingly, M2 macrophage markers, CD163, IL-6, and IL-10 also showed a significant increase in contrast to an insignificant increase in M1 markers (CD86 and iNOS). M2 macrophages secreted IL-6 and IL-10, but M1 macrophages did not. This result is concordant with a previous report, which revealed that M2 macrophages are the main contributor for IL-6 production in colon cancer [60]. In contrast, after treatment with M2 macrophage polarization inhibitors (O-ATP, trametinib, and bardoxolone methyl), there was a significant decrease in both EMR1 and M2 markers. By inhibiting IL-4-induced MEK/ERK signaling, trametinib specifically blocks M2-type polarization [61]. O-ATP is primarily a P2X7 antagonist, and P2X7 loss impairs TAM “M2-like” polarization [62]. Bardoxolone methyl, a bromodomain and extra-terminal protein and IkB kinase inhibitor, inhibits both M1- and M2-type polarization [61]. These macrophage polarization blockers also decrease EMR1 expression in cancer cells. Our co-culture results indicate that EMR1 expression is linked to macrophage polarization from monocytes, which eventually differentiate into TAMs.

In this study, we focused on the clinical significance of EMR1-TC in association with TAMs in CRC. Based on our findings, we concluded that EMR1 upregulation in cancer cells is regulated by TAM and has a significant relationship with lymph node metastasis, and the combined EMR1-TC^+^CD68^+^CD163^+^ expression can be used as a prognostic indicator in patients with CRC. Furthermore, EMR1 expression in colon cancer cells may be related to M2 macrophage polarization and vice versa. However, further research is warranted to investigate the signaling pathway between EMR1-TC and TAMs that involve tumor progression. This may help to clarify the biological role of EMR1 in colon cancer progression as well as reveal a new target for the treatment of patients with CRC.

## 5. Conclusions

We suggested that high EMR1-TC in CRC is associated with a high TAM expression. The combined EMR1-TC^+^CD68^+^CD163^+^ expression was significantly related to RFS in patients with CRC. The in vitro co-culture results indicated that EMR1 expression in colon cancer cells was linked to M2 macrophage polarization rather than M1 macrophage polarization, which may contribute to the LNM and CRC recurrence. In addition, macrophage polarization inhibitors can block EMR1 expression in colon cancer cells. Therefore, EMR1-TC can be used as a prognostic marker as well as a therapeutic target, especially for TAM-rich CRC.

## Figures and Tables

**Table 1:** Correlation between EMR1-TC and clinicopathologic parameters in MSI-H and MSS CRC.

Variables		EMR1-TC (Total)			EMR1-TC (MSI-H)			EMR1-TC (MSS)		
		N (%)		*p* Value	N (%)		*p* Value	N (%)		*p* Value
		Low	High		Low	High		Low	High	
**Gender**	Male	189 (47.5)	54 (13.6)	0.715	21 (29.6)	11 (15.5)	0.458	168 (51.4)	43 (13.1)	0.775
	Female	118 (29.6)	37 (9.3)		24 (33.8)	15 (21.1)		94 (28.7)	22 (6.7)	
**Location**	Right	120 (30.2)	52 (13.1)	0.003	33 (46.5)	23 (32.4)	0.113	88 (26.9)	29 (8.9)	0.112
	Left	187 (47.0)	39 (9.8)		12 (16.9)	3 (4.2)		174 (53.2)	36 (11.0)	
**T_stage**	Tis, T1, T2	43 (10.8)	21 (5.3)	0.039	2 (2.8)	5 (7.0)	0.044	41 (12.5)	16 (4.9)	0.088
	T3, T4	264 (66.3)	70 (17.6)		43 (60.6)	21 (29.6)		221 (67.6)	49 (15.0)	
**LNM**	LN^−^	187 (47.0)	39 (9.8)	0.003	38 (53.5)	12 (16.9)	0.001	149 (45.6)	27 (8.3)	0.037
	LN^+^	120 (30.2)	52 (13.1)		7 (9.9)	14 (19.7)		113 (34.6)	38 (11.6)	
**Lym_inv**	Not	169 (42.5)	40 (10.1)	0.073	29 (40.8)	10 (14.1)	0.030	140 (42.8)	30 (9.2)	0.332
	Yes	138 (34.7)	51 (12.8)		16 (22.5)	16 (22.5)		122 (37.3)	35 (10.7)	
**EMR1-SC**	Low	278 (69.8)	46 (11.6)	0.000	34 (47.9)	12 (16.9)	0.020	245 (74.9)	34 (10.4)	0.000
	High	29 (7.3)	45 (11.3)		11 (15.5)	14 (19.7)		17 (5.2)	31 (9.5)	
**CD163-SC**	Low	257 (64.7)	57 (14.4)	0.000	39 (54.9)	12 (16.9)	0.001	218 (66.9)	45 (13.8)	0.013
	High	49 (12.3)	34 (8.6)		6 (8.5)	14 (19.7)		43 (13.2)	20 (6.1)	
**CD68**	Low	204 (51.3)	31 (7.8)	0.000	19 (26.8)	3 (4.2)	0.008	185 (56.6)	28 (8.6)	0.000
	High	103 (25.9)	60 (15.1)		26 (36.6)	23 (32.4)		77 (23.5)	37 (11.3)	

Abbreviations: CD, cluster of differentiation; LNM, lymph node metastasis; Lym_inv, lymphatic invasion; N (%), number (percentage); SC, stroma cell; T_stage, tumor stage. *p* < 0.05 was considered statistically significant.

**Table 2:** Multivariate logistic regression analysis according to EMR1-TC (low vs. high) in MSI-H and MSS CRC.

Parameter	Total (MSI-H+MSS)				MSI-H				MSS			
	*p* Value	HR	95% C.I.		*p* Value	HR	95% C.I.		*p* Value	HR	95% C.I.	
			Lower	Upper			Lower	Upper			Lower	Upper
**LNM**	0.001	2.295	1.377	3.825	0.002	10.183	2.344	44.244	0.021	1.983	1.111	3.540
**CD68**	0.000	3.359	2.014	5.603	0.007	7.279	1.723	30.756	0.001	2.771	1.560	4.924
**CD163**	0.001	2.575	1.456	4.556	0.045	4.163	1.029	16.838	0.034	2.045	1.054	3.969

Abbreviations: C.I., confidence interval; CD, cluster of differentiation; CRC, colorectal cancer; EMR1-TC, EMR1 expression in tumor cells; HR, hazard ratio; LNM, lymph node metastasis; MSI-H, microsatellite unstable; MSS, microsatellite stable. *p*-value < 0.05 was considered statistically significant.

**Table 3:** Correlation between combined EMR1-TC^+^CD68^+^CD163^+^ expression and clinicopathologic parameters in MSI-H and MSS CRC.

Variables		(EMR1-TC^+^CD68^+^CD163^+^) Total (MSI-H+MSS)			(EMR1-TC^+^CD68^+^CD163^+^) (MSI-H)			(EMR1-TC^+^CD68^+^CD163^+^) (MSS)		
		N (%)		*p* Value	N (%)		*p* Value	N (%)		*p* Value
		Low	High		Low	High		Low	High	
**T_stage**	Tis, T1, T2	58 (14.6)	6 (1.5)	0.422	4 (5.6)	3 (4.2)	0.110	54 (16.5)	3 (0.9)	0.114
	T3, T4	289 (72.6)	45 (11.3)		54 (76.1)	10 (14.1)		235 (71.9)	35 (10.7)	
**LNM**	LN-	207 (52.0)	19 (4.8)	0.004	45 (63.4)	5 (7.0)	0.015	162 (49.5)	14 (4.3)	0.037
	LN+	140 (35.2)	32 (8.0)		13 (18.3)	8 (11.3)		127 (38.8)	24 (7.3)	
**Lym_inv**	Not	192 (48.2)	17 (4.3)	0.004	37 (52.1)	2 (2.8)	0.002	155 (47.4)	15 (4.6)	0.121
	Yes	155 (38.9)	34 (8.5)		21 (29.6)	11 (15.5)		134 (41.0)	23 (7.0)	
**Recurrence or not**	Not	311 (78.3)	20 (5.0)	0.000	53 (74.6)	8 (11.3)	0.014	258 (79.1)	12 (3.7)	0.000
	Yes	35 (8.8)	31 (7.8)		5 (7.0)	5 (7.0)		30 (9.2)	26 (8.0)	

Abbreviations: CD, cluster of differentiation; EMR1-TC, EMR1 expression in tumor cells; LNM, lymph node metastasis; Lym_inv, lymphatic invasion; MSI-H, microsatellite unstable; MSS, microsatellite stable; N (%), number (percentage); T_stage, tumor stage. *p* < 0.05 was considered statistically significant.

**Table 4:** Univariate and multivariate analyses of the prognostic factors in patients with CRC using a Cox regression model.

Parameters	Recurrence Free Survival (RFS)							
	Univariate Analysis				Multivariate Analysis			
	*p*-Value	HR	95.0% CI		*p*-Value	HR	95.0% CI	
			Lower	Upper			Lower	Upper
**T_Stage (l-ll vs lll-lV)**	0.008	14.776	2.050	106.515	0.023	9.981	1.365	73.015
**LNM (LN^+^ vs LN^−^)**	0.000	2.659	1.609	4.394	0.229	1.405	0.807	2.447
**Lymph_inv (No vs Yes)**	0.000	2.662	1.597	4.436	0.077	1.657	0.947	2.900
**EMR1-TC (Low vs High)**	0.843	0.940	0.512	1.728				
**EMR1-SC (Low vs High)**	0.446	0.769	0.392	1.509				
**CD68 (Low vs High)**	0.070	0.610	0.357	1.041				
**CD163-SC (Low vs High)**	0.241	0.678	0.354	1.298				
**Combined EMR1-TC^+^CD68^+^CD163^+^ (Low vs High)**	0.000	8.169	5.011	13.318	0.000	6.565	3.988	10.806

Abbreviations: CD, cluster of differentiation; C.I., confidence interval; EMR1-SC, EMR1 expression in stroma cells; EMR1-TC, EMR1 expression in tumor cells; HR, hazard ratio; LNM, lymph node metastasis; Lym_inv, lymphatic invasion; T_stage, tumor stage. *p*-value < 0.05 was considered statistically significant.

## Data Availability

Not applicable.

## Supplementary Materials

The following supporting information can be downloaded at: https://www.mdpi.com/article/10.3390/biomedicines10123121/s1, Table S1: Spearman’s rho Correlation analysis (bivariate) of EMR1, CD68, and CD163 with LNM in MSI-H, MSS CRC. Table S2: Univariate and multivariate analyses of the prognostic factors in patients with CRC using a Cox regression model. Figure S1: High EMR1 activation in colorectal cancer cells was induced by macrophages. Figure S2: Toxicity of M2-type macrophage polarization inhibitors (O-ATP, trametinib, Bardoxolone methyl) in colon cancer cells (HCT15, Caco2) and myeloid cells (THP-1 monocyte).
