# Supplementary material for: EMR1/ADGRE1 Expression in Cancer Cells Upregulated by Tumor-Associated Macrophages Is Related to Poor Prognosis in Colorectal Cancer
Source: Biomedicines. 2022 Dec 2;10(12):3121. doi: 10.3390/biomedicines10123121 (PMC9775542; doi:10.3390/biomedicines10123121)
Supplement: Supplementary file 1 [file biomedicines-10-03121-s001.zip › biomedicines-2028976-supplementary.pdf]

biomedicines

MDPI

Supplementary Materials

EMR1/ADGRE1 Expression in Cancer Cells Upregulated by Tumor-Associated Macrophages Is Related to Poor Prognosis in Colorectal Cancer

Supplementary file

Table S1. Spearman's rho Correlation analysis (bivariate) of EMR1, CD68, and CD163 with LNM in MSI-H, MSS CRC.

	Correlation Coefficient						
**Variables**	**MSI_Type**	**LN**	**MUC5AC**	**EMR1_TC**	**EMR1_SC**	**CD68**	**CD163_SC**
**CRC (MSI-H+MSS)**							
LNM	.127*	--		.153**			
EMR1_TC	-.153**	.153**	.203**	--	.432**	.283**	.221**
EMR1_SC	-.216**		.186**	.432**	--	.244**	.152**
CD68	-.114*			.283**	.244**	--	.254**
CD163_SC				.221**	.152**	.254**	--
**MSI-H CRC**							
LNM		..		.404**			
EMR1_TC		.404**	.285*	..	.297*	.420**	.434**
EMR1_SC				.297*	..		
CD68				.420**		..	.447**
CD163_SC				.434**		.447**	..
**MSS CRC**							
LNM		..		.123*			-.109*
EMR1_TC		.123*		..	.465**	.231**	.145**
EMR1_SC				.465**	..	.241**	.125*
CD68				.231**	.241**	..	.195**
CD163_SC		-.109*		.145**	.125*	.195**	..

*. Correlation is significant at the $p < 0.05$ (2-tailed). **. Correlation is significant at the $p < 0.05$ (2-tailed).

Table S2. Univariate and multivariate analyses of the prognostic factors in patients with CRC using a Cox regression model.

Parameters	Overall survival (OS)							
	Univariate analysis				Multivariate analysis			
	P-value	HR	95 % CI		*P*-value	HR	95 % CI	
			Lower	Upper			Lower	Upper
**T_Stage (l-ll vs lll-lV)**	.198	2.182	.665	7.152	.140	2.522	.738	8.610
**LNM (LN+ vs LN-)**	.642	1.177	.592	2.340	.732	0.870	.392	1.929
**Lymph_inv (No vs Yes)**	.217	1.542	.776	3.064	.370	1.420	.660	3.055
**EMR1_TC (Low vs High)**	.161	1.702	.809	3.584	.157	1.924	.777	4.762
**EMR1_SC (Low vs High)**	.343	1.470	.663	3.261	.656	1.228	.496	3.040
**CD68 (Low vs High)**	.386	0.720	.342	1.513	.164	0.562	.250	1.265
**CD163_SC (Low vs High)**	.681	1.182	.533	2.621	.593	1.263	.537	2.970
**Combined EMR1TC^{+}CD68^{+}CD163^{+} (Low vs High)**	.790	1.216	.288	5.135	.993	1.005	.328	3.078

Abbreviations: CD, cluster of differentiation; C.I., confidence interval; EMR1-SC, EMR1 expression in stroma cells; EMR1-TC, EMR1 expression in tumor cells; HR, hazard ratio; LNM, lymph node metastasis; Lym_inv, lymphatic invasion; T_stage, tumor stage. *p*-value < 0.05 was considered statistically significant.

A

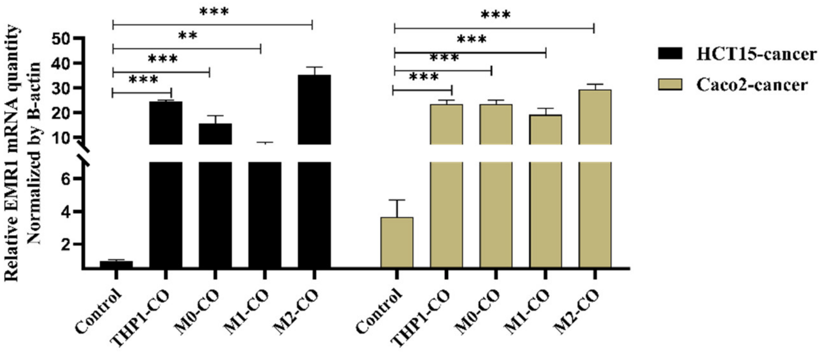

Figure S1. High EMR1 activation in colorectal cancer cells was induced by macrophages. (**A**) Relative EMR1 mRNA level was detected in colon cancer cells (HCT15, Caco2) after being co-cultured with myeloid cells (THP-1monocyte, M0, M1, M2 macrophages) for 48 h as determined by real-time quantitative reverse transcription polymerase chain reaction. Results were normalized by B-actin. Error bars denote standard deviation. *Statistically significant at *$p < 0.033$; **$p < 0.002$; ***$p < 0.001$ in comparison with the control group via multiple Bonferroni two-way ANOVA group test.

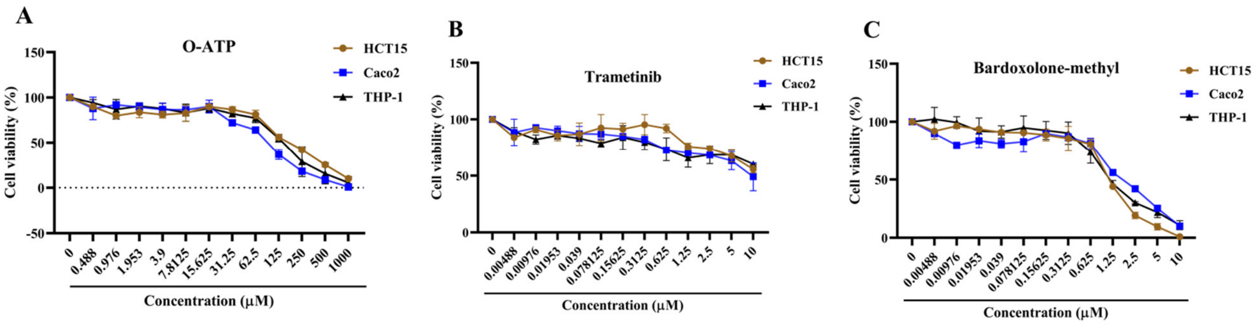

Figure S2. Toxicity of M2-type macrophage polarization inhibitors (O-ATP, trametinib, Bardoxolone methyl) in colon cancer cells (HCT15, Caco2) and myeloid cells (THP-1 monocyte). (**A–C**) Cells were treated with O-ATP, trametinib, and Bardoxolone methyl for 48 h, and toxicity was detected using a WST-1 reagent. Graphs represent data as means ± standard deviation.
